# Supplementary figures and images for: Adaptive and maladaptive expression plasticity underlying herbicide resistance in an agricultural weed
Source: Evol Lett. 2021 Jun 14;5(4):432–40. doi: 10.1002/evl3.241 (PMC8327940; doi:10.1002/evl3.241)

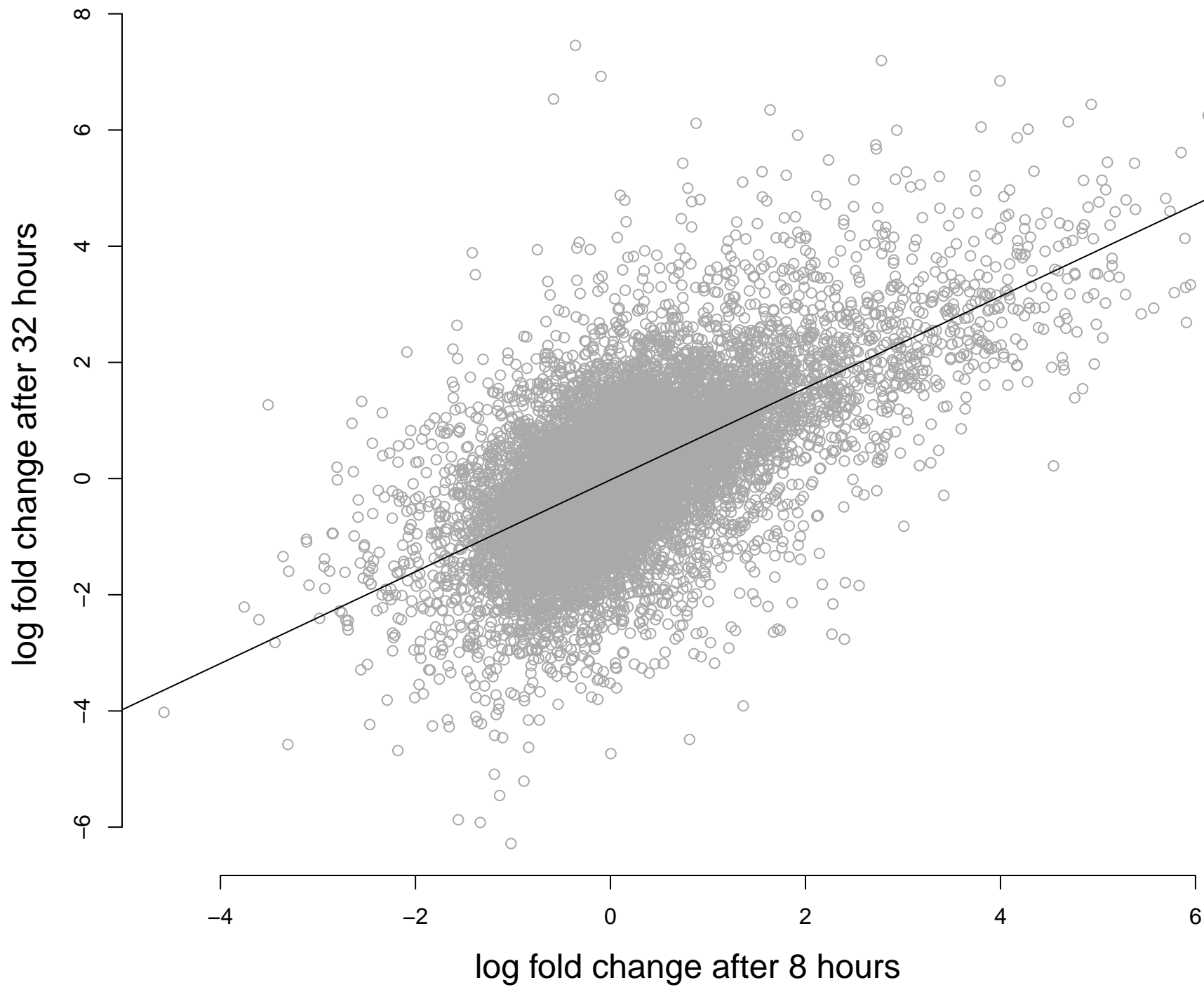

Supplement: Supplementary file 1 — Figure S1: Plastic responses to herbicide spray 8 and 32 hours after treatment. The x axis shows the log‐fold difference in expression in sprayed and unsprayed conditions 8 hours after treatment and the y axis shows the same value 32 hours after treatment. The black dotted line shows a line with a slope of 1 and intercept of 0. The correlation coefficient is 0.616. All genes are shown. [file EVL3-5-432-s003.pdf]

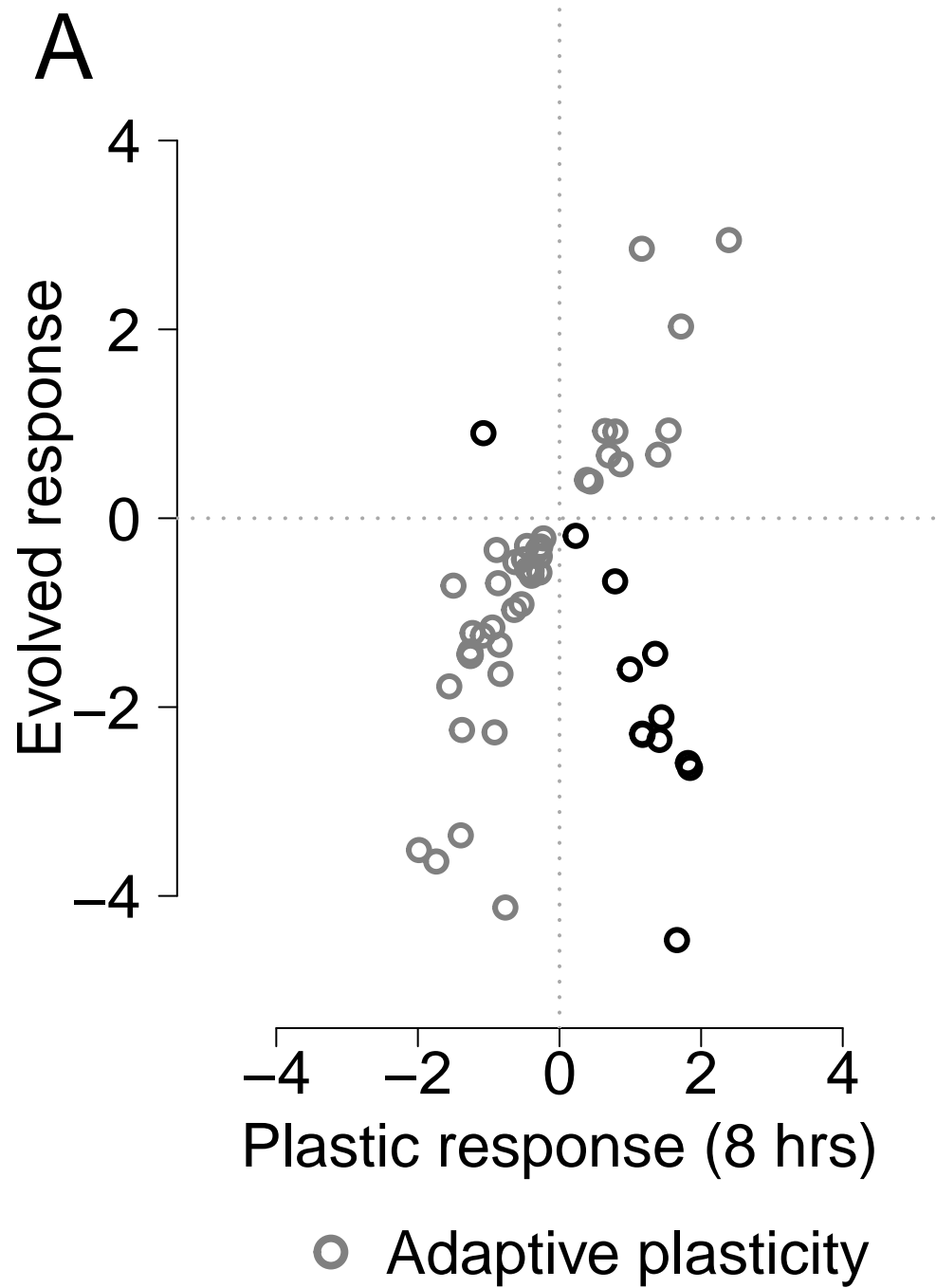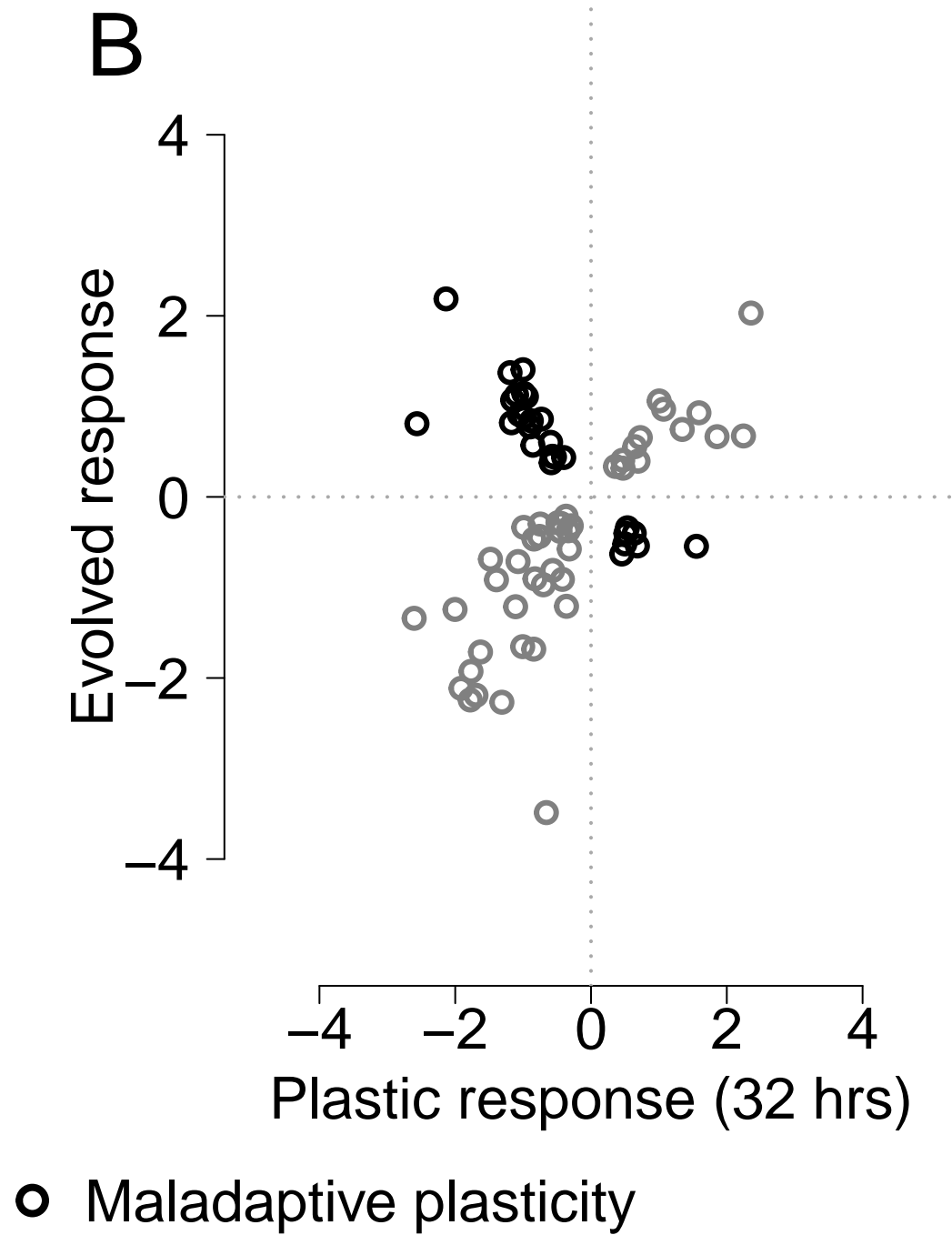

Supplement: Supplementary file 2 — Figure S2: Adaptive and maladaptive expression plasticity at 8 hours (A) and 32 hours (B) after herbicide treatment. In both panels, the x axis shows plastic responses to glyphosate, specifically the log fold change in expression between sprayed and nonsprayed conditions where positive values indicate increased expression in herbicide spray. The y axis shows the log fold difference in expression between lines selected for increased resistance to herbicide and lines that were not selected (‘original lines’), where positive values indicate increased expression in the resistance selection lines. Each point represents a gene and all genes where there was both a significant plastic and an evolved response are shown (FDR < 0.1). [file EVL3-5-432-s004.pdf]

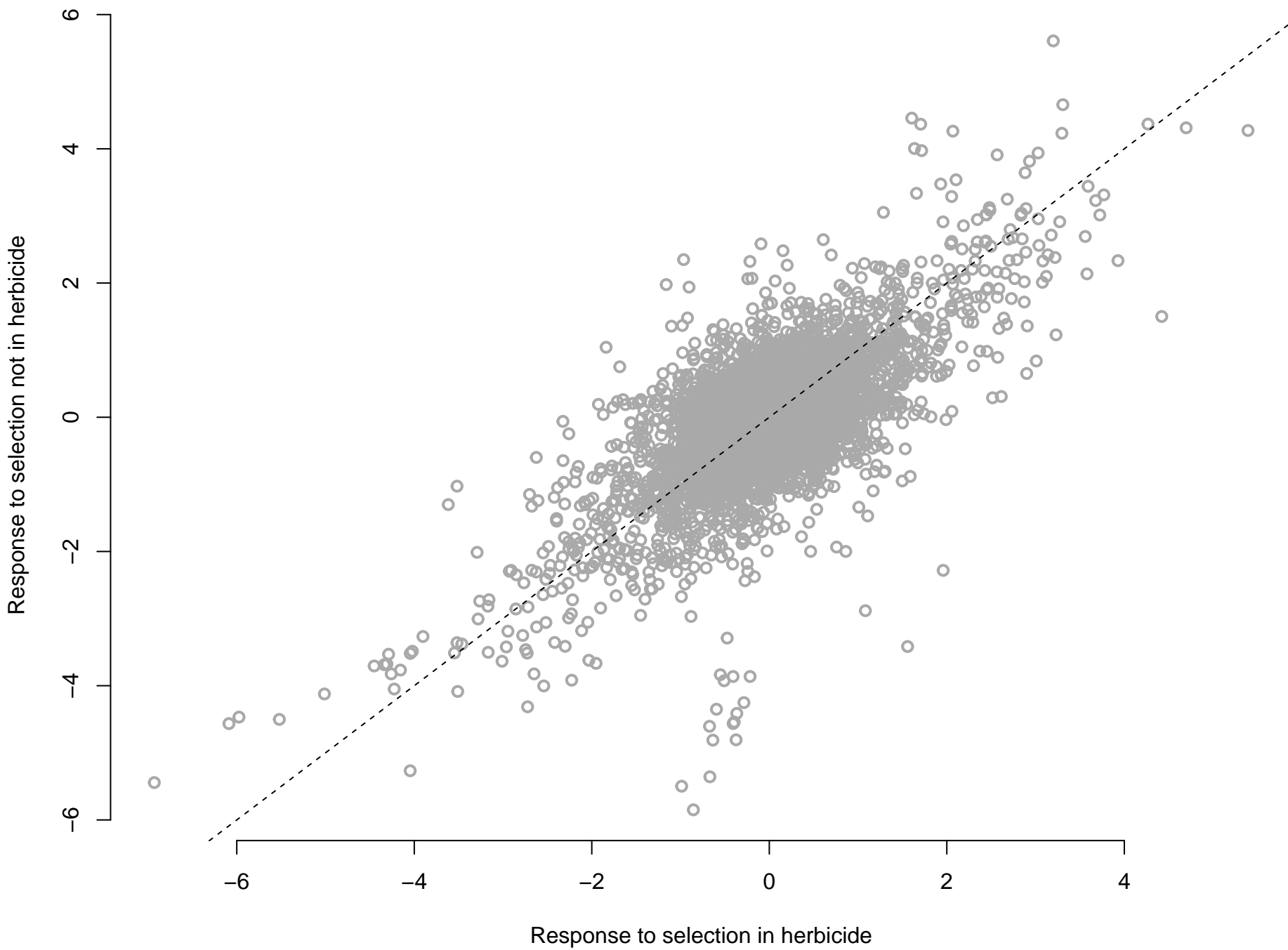

Supplement: Supplementary file 3 — Figure S3: Plastic expression changes in control and resistance lines for all genes. Each point represents one gene, and the y = x line is plotted with a dashed line. [file EVL3-5-432-s001.pdf]
